# Knowledge, attitudes, practices, and influencing factors associated with chronic rhinosinusitis among healthcare trainees and professionals in Hail, Saudi Arabia: a principal component analysis

**DOI:** 10.3389/fpubh.2026.1808606

**Published:** 2026-08-14

**Authors:** Abdullah D. Alotaibi, Abdulaziz S. Alqahtani, Mohammed Fahad Alateeq, Mohd Saleem, Hatim Salem Alanzi, Robah Abdulaziz Alshuhail, Hamad Rudhayman Alrashdi, Osama Abdullah Almuzaini, Sheeba Afreen, Tahani N. Almofeed Altamimi, Azharuddin Sajid Syed Khaja

**Affiliations:** 1 Department of Otolaryngology, College of Medicine, University of Hail, Hail, Saudi Arabia; 2 Department of Pathology, College of Medicine, University of Hail, Hail, Saudi Arabia; 3 College of Medicine, University of Hail, Hail, Saudi Arabia; 4 Department of Biochemistry, Career Institute of Medical Science and Hospital, Lucknow, Uttar Pradesh, India; 5 Department of Family and Community Medicine, College of Medicine, University of Hail, Hail, Saudi Arabia

**Keywords:** attitudes, chronic rhinosinusitis, healthcare workers, knowledge, practices, principal component analysis

## Abstract

**Background and objectives:**

Chronic rhinosinusitis (CRS) imposes substantial clinical and economic burdens worldwide. In Saudi Arabia, prevalence estimates exceed 20% among adults, yet limited data are available regarding awareness, knowledge, attitudes, and practices (A-KAP) related to CRS among current and future healthcare providers. This study evaluated A-KAP regarding CRS among healthcare trainees and a smaller subgroup of healthcare professionals in the Hail region of Saudi Arabia and identified sociodemographic factors associated with CRS knowledge.

**Methods:**

A cross-sectional survey (Google Forms) was conducted among 363 participants using a structured questionnaire comprising 59 A-KAP items. Descriptive statistics, chi-square tests, and binary logistic regression were used to identify sociodemographic factors associated with CRS knowledge. Principal component analysis (PCA) was performed to explore latent knowledge domains (*α* = 0.05).

**Results:**

Overall, 73.0% of participants heard of CRS. Nasal congestion (90.9%) and nasal discharge (68.6%) were the most recognized symptoms, whereas orbital abscess (12.9%) and genetic predisposition (0.3%) were the least recognized. Arabic speakers demonstrated greater CRS awareness than English speakers (AOR = 0.21, 95% CI: 0.06–0.69), and participants with monthly incomes of 5,000–15,000 SAR were more likely to demonstrate good CRS knowledge than higher-income participants (AOR = 2.88, 95% CI: 1.21–6.86). PCA identified distinct symptoms, risk factors, and complication knowledge domains. Participants generally demonstrated positive attitudes toward healthcare-seeking behavior, specialist follow-up, and public awareness; however, uncertainty regarding treatment effectiveness (45.5%) and openness toward alternative or herbal therapies (28.4%) persisted.

**Conclusion:**

Although participants demonstrated moderate awareness and generally positive attitudes toward CRS, important knowledge gaps remained, particularly regarding complications and genetic risk factors. Several sociodemographic factors were associated with CRS knowledge, highlighting the need for targeted educational interventions to strengthen CRS-related knowledge among healthcare trainees and professionals.

## Introduction

Chronic rhinosinusitis (CRS) is a common and persistent inflammatory disease of the nasal cavity and paranasal sinuses that substantially impairs quality of life and imposes a significant clinical and economic burden. Patients with CRS often experience prolonged symptoms, reduced productivity, and recurrent healthcare utilization, and many require long-term medical therapy or surgical intervention, including functional endoscopic sinus surgery (FESS) (1–3). Effective CRS management, therefore, depends not only on specialist care but also on early recognition, appropriate referral, and patient education by healthcare professionals across different levels of the healthcare system. In this context, awareness, knowledge, attitudes, and practices (A-KAP) related to CRS among health sciences students and healthcare workers play a critical role in improving clinical outcomes and reducing disease burden.

According to internationally accepted guidelines, including the European Position Paper on Rhinosinusitis and Nasal Polyps (EPOS 2020) and the EUFOREA consensus statements, CRS is defined as inflammation of the nose and paranasal sinuses persisting for at least 12 weeks and characterized by the presence of two or more symptoms, one of which must be nasal obstruction or nasal discharge, with or without facial pain/pressure and/or a reduction or loss of smell (4–6). The inclusion of these core symptoms is essential for establishing a CRS diagnosis and guiding clinical evaluation, highlighting the importance of recognizing symptoms among healthcare professionals. Recognition of these cardinal symptoms is essential for establishing a clinical diagnosis and guiding further evaluation. However, CRS is a heterogeneous condition with a multifactorial pathophysiology involving epithelial dysfunction, microbial factors, allergic inflammation, and environmental exposures, which complicates diagnosis and management and requires a broad and integrated level of clinical understanding (2, 7). The economic impact of CRS is considerable, with estimates from the United States indicating billions of dollars annually in direct and indirect costs, further underscoring the importance of informed and timely clinical decision-making (8).

Despite the availability of clear diagnostic criteria and evidence-based management guidelines, delayed recognition and incomplete understanding of CRS remain common, particularly outside specialized otolaryngology settings. Limited awareness of less specific symptoms, risk factors, and potential complications may contribute to underdiagnosis, delayed referral, and suboptimal management. Healthcare trainees and early-career providers represent a particularly important group in this regard, as they are future frontline clinicians responsible for early symptom recognition, patient counseling, and referral decisions. In addition, occupational and environmental exposures, such as airborne irritants and pollutants, are recognized as modifiable risk factors for CRS, highlighting the need for healthcare providers to integrate environmental and occupational assessments into routine practice (9).

The CRS management is further complicated by frequent comorbidities, including allergic rhinitis and immune-mediated conditions, which often require multidisciplinary evaluation and coordinated care (10, 11). Moreover, disparities in CRS incidence and outcomes across populations emphasize the influence of sociodemographic and sociocultural factors on disease presentation and management (12). Together, these complexities underscore the importance of comprehensive education and training strategies to strengthen A-KAP among healthcare workers and health sciences students, thereby improving patient outcomes and supporting public health initiatives (13, 14).

In Saudi Arabia, CRS represents a substantial public health concern, with prevalence estimates higher than those reported in many Western populations, particularly among older individuals and those with chronic illnesses (15). This high disease burden underscores the need for targeted educational interventions to improve awareness, diagnostic accuracy, and clinical skills related to CRS management (16). Sociodemographic characteristics, such as age, gender, education, and employment status, are known to influence health-related knowledge and behaviors, and emerging evidence suggests that knowledge gaps may persist among healthcare professionals despite formal training, particularly in areas related to infection control and sinus disease prevention (17, 18). Furthermore, CRS is shaped by an interplay of biological, genetic, environmental, and sociocultural factors, which reinforces the need for a comprehensive and context-sensitive approach to education and practice (19–22).

Recent regional studies have begun to clarify the epidemiological and clinical profile of CRS in Saudi Arabia. A national systematic review of 14 studies involving 12,667 patients reported that CRS without nasal polyps was the most common subtype, followed by allergic fungal rhinosinusitis, with nasal obstruction and rhinorrhea as the predominant symptoms and the maxillary sinus most frequently affected (23). The review also highlighted substantial regional variation in clinical presentation and management, underscoring the influence of geographic and sociocultural factors and the need for population-specific awareness and educational strategies. Complementing these findings, a tertiary-care study from Riyadh involving 660 patients found that 67.7% of CRS cases had nasal polyps, with high rates of comorbid asthma and aspirin hypersensitivity, particularly among older patients and those with environmental allergies (24). Together, these studies illustrate the heterogeneous CRS phenotypes observed in Saudi Arabia and emphasize the importance of improving recognition of associated comorbidities and systemic features in clinical practice.

Despite the recognized burden of CRS in Saudi Arabia, data on CRS-related A-KAP among health sciences students and healthcare workers at the regional level remain limited, particularly outside major metropolitan centers. The Hail region provides a relevant setting for examining these issues, given its demographic profile and healthcare system. Understanding current awareness patterns and identifying knowledge gaps in this population may inform targeted educational strategies and curriculum development to improve early recognition and appropriate management of CRS.

Therefore, this study aimed to assess awareness, knowledge, attitudes, and practices related to chronic rhinosinusitis among healthcare trainees and professionals in the Hail region of Saudi Arabia. In addition, the study aimed to identify sociodemographic factors associated with participants’ knowledge of CRS using logistic regression analysis.

## Materials and methods

### Study design

A quantitative, descriptive, analytical cross-sectional study design was used to assess A-KAP levels related to CRS among healthcare trainees and professionals.

### Study population and sampling technique

The study population comprised male and female participants aged 18–65 years who were actively engaged in health-related education or professional practice. Eligible participants were recruited via convenience sampling. The sample included students enrolled in medical and other health-related programs, together with a smaller subgroup of practicing healthcare professionals.

Inclusion criteria required that participants be 18 years of age or older, currently enrolled in or employed in a healthcare-related field, and willing to provide informed consent. Individuals younger than 18 years, those not involved in healthcare education or practice, or those unwilling to provide consent were excluded. The sample size was calculated using the Raosoft^®^ online sample size calculator, assuming a 5% margin of error, a 95% confidence interval, and a 50% response distribution. After accounting for potential non-response, a final sample of 363 participants was obtained. Although the achieved sample size was slightly below the estimated target for large populations, it was considered adequate for exploratory cross-sectional analyses within the study setting. Given the substantial representation of health sciences students, the study was designed to capture awareness patterns among both future and practicing healthcare providers.

### Questionnaire development and validation

A structured, self-administered questionnaire was developed in both English and Arabic and distributed electronically via Google Forms to collect participant data. Questionnaire items were developed based on a comprehensive review of the literature on CRS epidemiology, diagnosis, and management, including international clinical guidelines (EPOS 2020 and EUFOREA consensus statements) and prior survey-based KAP studies in respiratory and otolaryngologic conditions (25–27). Content validity was assessed by a panel of three otolaryngology faculty members, who independently reviewed all items for clarity, relevance, and coverage of key CRS constructs. Based on their feedback, minor revisions were made to refine wording, eliminate redundancy, and ensure adequate representation of symptoms, risk factors, complications, and attitude/practice domains.

The final questionnaire consisted of five sections. Section 1 collected demographic and socioeconomic information, including age, gender, education level, residence, and income. Section 2 assessed CRS-related knowledge, focusing on symptoms, risk factors, and complications. Section 3 explored attitudes toward CRS, including perceived severity, treatment options, and lifestyle influences. Section 4 examined perceptions and beliefs related to CRS and its management, and Section 5 identified sources of CRS-related information. Most items were closed-ended and presented as multiple-choice or Likert-scale questions, with a limited number of open-ended items included to capture additional qualitative insights.

Prior to the main study, the questionnaire was pilot-tested among 30 health sciences students and healthcare workers to assess clarity, comprehensibility, and completion time. Internal consistency reliability of the knowledge items was assessed using Cronbach’s alpha, which yielded a value of 0.82, indicating good internal consistency. The instrument was developed specifically for this study and informed by established literature and clinical guidelines.

In addition, a knowledge-scoring system was used to quantify participants’ understanding of CRS. Each correct response in the knowledge section was assigned a score of 1, while incorrect or “do not know” responses were assigned a score of 0. Open-ended responses were not included in knowledge scoring. The total knowledge score for each participant was calculated by summing the item scores. Based on the distribution of scores, participants were categorized into “good knowledge” and “poor knowledge” groups using the median score as the cut-off point.

### Statistical analysis

Data were analyzed using Microsoft Excel and SPSS software (version 23.0; IBM Corp., Armonk, NY, United States). Descriptive statistics, including frequencies and percentages, were used to summarize the sociodemographic characteristics and A-KAP variables. Associations between CRS knowledge and demographic factors were assessed using chi-square (*χ*^2^) tests. Binary logistic regression analysis was performed to identify independent sociodemographic predictors of CRS knowledge.

Principal component analysis (PCA) was performed as an exploratory technique to identify latent knowledge domains among CRS symptom, risk-factor, and complication items (Supplementary Tables S1–S3). Prior to PCA, data suitability was assessed using the Kaiser–Meyer–Olkin (KMO) measure of sampling adequacy and Bartlett’s test of sphericity. Components with eigenvalues greater than 1.0 were retained. A two-sided *p*-value < 0.05 was considered statistically significant. Variables identified as significant in the *χ*^2^ analyses were subsequently included in multivariable logistic regression models to estimate adjusted associations.

## Results

### Sociodemographic details of the study participants

**Figure 1:**
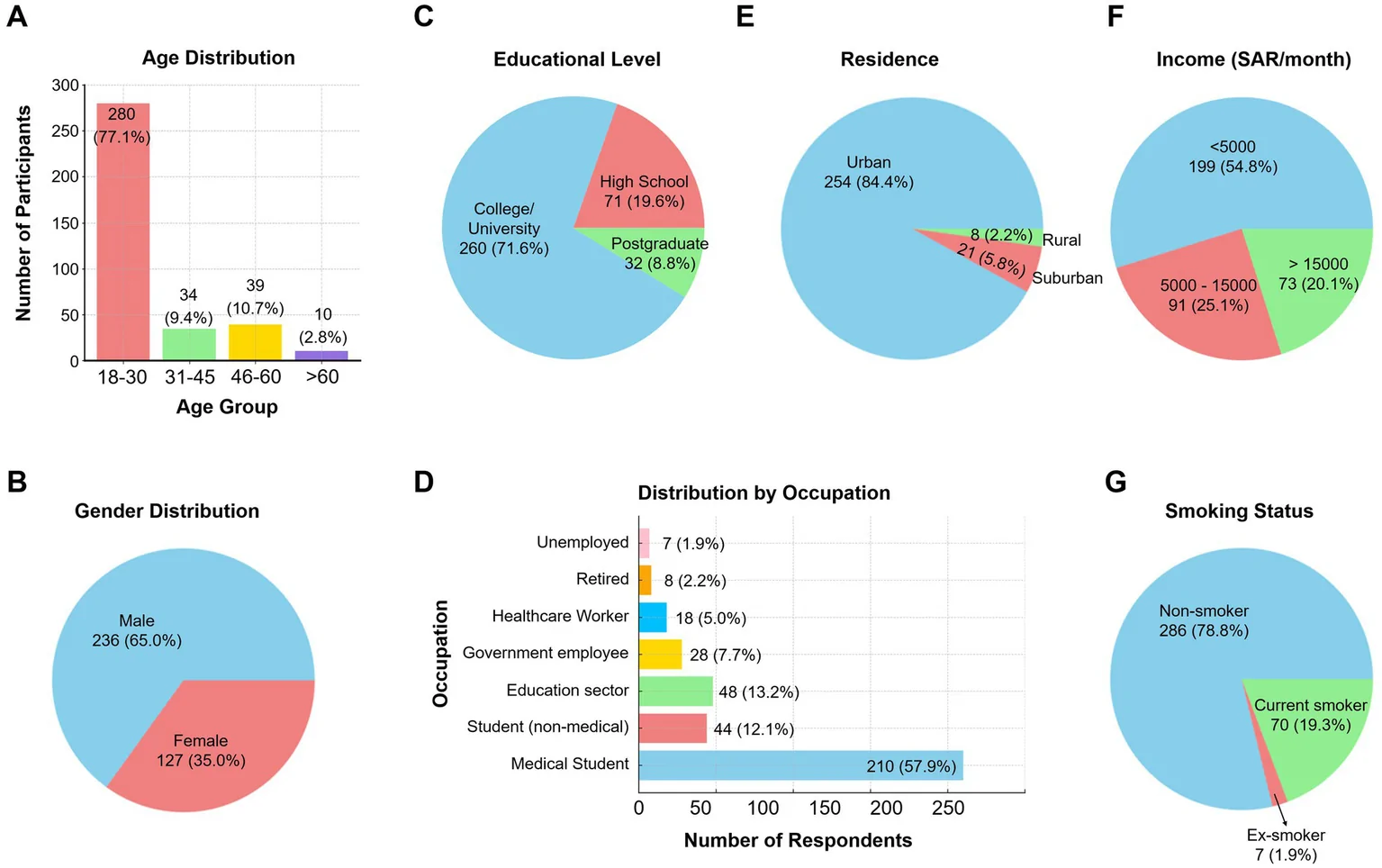
Sociodemographic characteristics of the study population (*N* = 363). **(A)** Age distribution of the participants. **(B)** Gender distribution. **(C)** Educational attainment level. **(D)** Distribution of participants by occupation. **(E)** Geographic residence of the participants. **(F)** Monthly income level in SAR (Saudi Arabian Riyals). **(G)** Smoking status of the cohort. The values represent counts with percentages.

The distribution of respondents based on sociodemographic variables (Figure 1) showed that most were between 18 and 30 years old (77.1%, Figure 1A), followed by those aged 46–60 years (10.7%), 31–45 years (9.4%), and over 60 years (2.8%). Regarding sex, 65.0% of respondents were male, and 35.0% were female (Figure 1B).

Regarding educational level (Figure 1C), a significant proportion of participants (71.6%) had completed college or university, followed by high school graduates (19.6%) and postgraduates (8.8%). In terms of occupation (Figure 1D), the largest group was students in the health sciences (57.9%), followed by those working in education (13.2%), non-medical students (12.1%), government employees (7.7%), healthcare workers (5.0%), retired individuals (2.2%), and unemployed individuals (1.9%). In our study, only 5.0% of respondents were practicing healthcare workers. Therefore, inferential comparisons between healthcare professionals and other occupational groups (e.g., students, non-medical occupations) were not statistically robust and should be interpreted with caution.

Concerning place of residence, most participants lived in urban areas (92.0%), with 5.8% residing in suburban regions and 2.2% in rural areas (Figure 1E). Regarding monthly income, 54.8% earned less than 5,000 SAR, 25.1% earned between 5,000 and 15,000 SAR, and 20.1% earned more than 15,000 SAR (Figure 1F). As for smoking status, the majority of respondents were nonsmokers (78.8%), with 19.3% being current smokers and 1.9% ex-smokers (Figure 1G).

### Knowledge about CRS among the study participants

**Figure 2:**
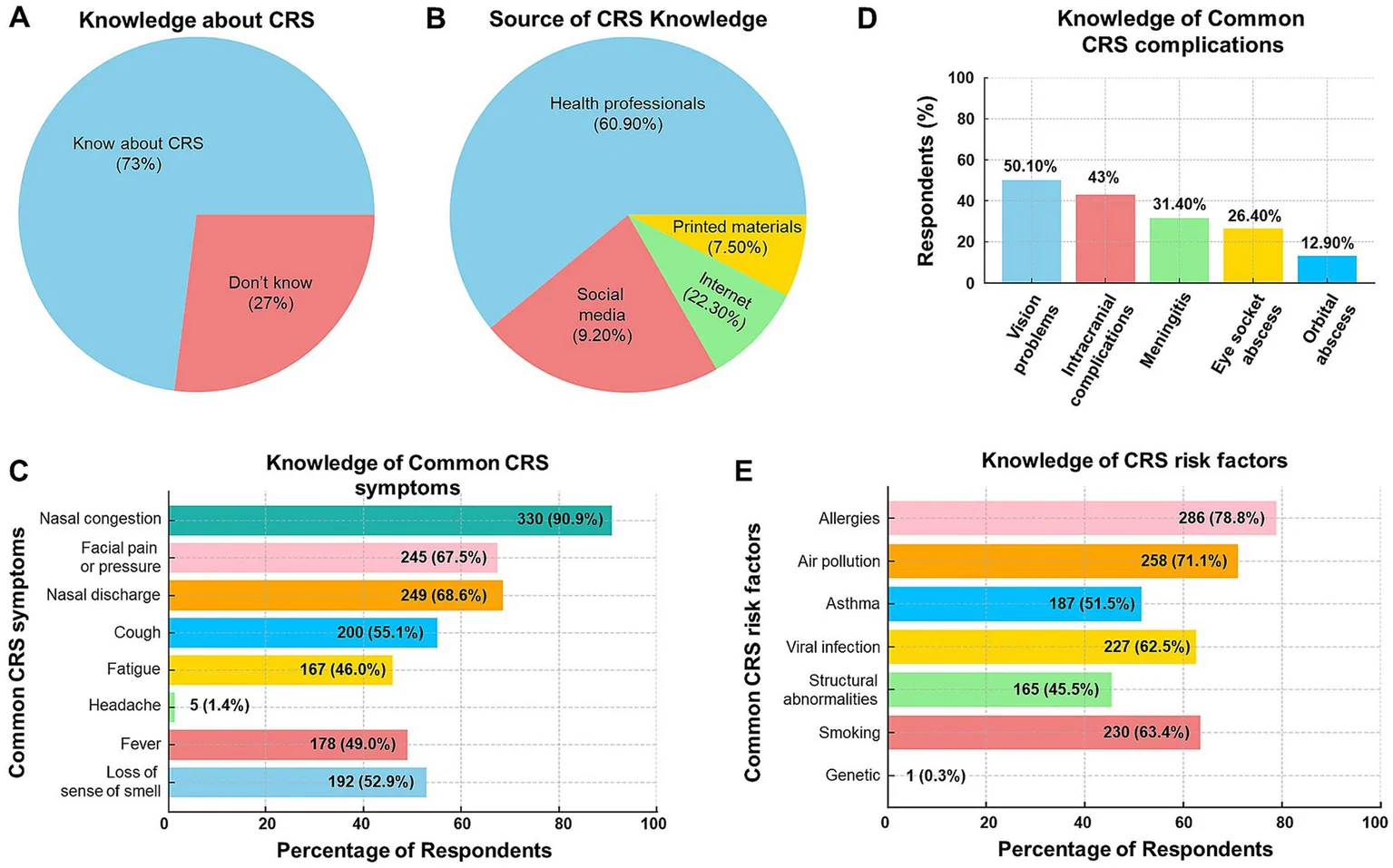
Knowledge regarding chronic rhinosinusitis (CRS) among the study participants. **(A)** Awareness about CRS: 73% of the respondents reported knowing about CRS, whereas 27% were unaware. **(B)** Sources of CRS knowledge: The majority obtained information from health professionals (60.9%), followed by the internet (22.3%), social media (9.2%), and printed materials (7.5%). **(C)** Knowledge of common CRS symptoms: Nasal congestion (90.9%) was the most recognized symptom, followed by nasal discharge (68.6%) and facial pain or pressure (67.5%). **(D)** Knowledge of common CRS complications: Vision problems (50.1%) and intracranial complications (43%) were the most frequently recognized complications. **(E)** Knowledge of CRS risk factors: Allergies (78.8%) and air pollution (71.1%) were the most frequently identified.

Figure 2 demonstrates the respondents’ knowledge of CRS, including symptoms, risk factors, complications, and sources of information. Regarding awareness of CRS, most respondents (73.0%) reported familiarity with the condition, while 27.0% are not (Figure 2A). The majority (60.9%) identified health professionals as their main source of information about CRS (Figure 2B). The internet was cited by 22.3%, followed by social media (9.2%) and printed materials such as books and pamphlets (7.5%). This indicates that professional healthcare advice remains the most trusted and common source of CRS information, although digital platforms are increasingly influencing public awareness.

The respondents exhibited varying levels of awareness of CRS complications (Figure 2C). The most commonly recognized complication was vision problems, identified by 50.1% of participants. 43.0% recognized intracranial complications, while 31.4% recognized that meningitis could be a potential consequence of CRS. Eye socket abscesses were identified by 26.4%, and orbital abscesses were the least recognized, with only 12.9% acknowledging them. This indicates a moderate awareness of serious CRS complications among the surveyed group.

The distribution of respondents by knowledge of common CRS symptoms showed that the most recognized symptom was nasal congestion, reported by 90.9% of participants (Figure 2D). This was followed by nasal discharge (68.6%) and facial pain or pressure (67.5%). Other frequently reported symptoms included cough (55.1%), loss of smell (52.9%), fever (49.0%), and fatigue (46.0%). Notably, only a small percentage of respondents (1.4%) identified headaches as a CRS symptom.

The respondents’ knowledge of the risk factors for CRS (Figure 2E) showed that allergies were the most common, reported by 78.8% of participants. This was followed by air pollution (71.1%), smoking (63.4%), and viral infections (62.5%). Asthma was recognized as a risk by 51.5%, while 45.5% were aware that structural abnormalities, such as a deviated septum, could contribute. Only a very small percentage (0.3%) identified genetic factors as risks for CRS.

### Sociodemographic influencing factors associated with CRS knowledge

Several sociodemographic influencing factors were significantly associated with CRS knowledge (Table 1). Language was significantly associated with CRS awareness (*χ*^2^ = 5.506, *p* = 0.019), with more Arabic speakers (74.1%) than English speakers (46.7%) showing awareness. Education level also showed a significant relationship (*χ*^2^ = 12.396, *p* = 0.002), with individuals holding a high school diploma demonstrating the highest level of knowledge (87.3%), followed by those with college or university degrees (71.2%), and the lowest level of knowledge among postgraduates (56.3%).

**Table 1:** Association between CRS knowledge and sociodemographic variables among study participants.

Variable	Knowledge regarding CRS		Significance	
	No (%)	Yes (%)	Chi sq	*p*-value
Language				
English	8 (53%)	7 (47%)	5.506	**0.019**
Arabic	90 (26%)	258 (74%)		
Age				
18–30 years	74 (26.4%)	206 (73.6%)	2.368	0.500
31–45 years	7 (21%)	27 (79%)		
46–60 years	14 (36%)	25 (64%)		
More than 60 years	3 (30%)	7 (70%)		
Gender				
Male	62 (26%)	174 (74%)	0.18	0.671
Female	36 (28%)	91 (72%)		
Educational level				
High school	9 (13%)	62 (87%)	12.396	**0.002**
College/University	75 (29%)	185 (71%)		
Postgraduate	14 (44%)	18 (56%)		
Occupation				
Students in the health field	53 (25%)	157 (75%)	13.139	**0.041**
Students (nonmedical)	12 (27%)	32 (73%)		
Education sector Job	9 (19%)	39 (81%)		
Government employee	15 (54%)	13 (46%)		
Healthcare worker	4 (22%)	14 (78%)		
Retired	2 (25%)	6 (75%)		
Unemployed	3 (43%)	4 (57%)		
Place of residence				
Urban	82 (25%)	252 (75%)	12.845	**0.002**
Suburban	12 (57%)	9 (43%)		
Rural	4 (50%)	4 (50%)		
Monthly income (in SAR)				
Less than 5,000 per month	59 (30%)	140 (70%)	10.922	**0.004**
5,000–15,000 per month	13 (14%)	78 (86%)		
More than 15,000 per month	26 (36%)	47 (64%)		

Bold values indicate statistically significant results (*p* < 0.05).

The participants’ occupation was another variable significantly associated with CRS knowledge (*χ*^2^ = 13.139, *p* = 0.041). Those working in the education sector (81.3%) and the health sciences (74.8%) showed higher awareness than government employees (46.4%) and unemployed individuals (57.1%). Place of residence was also significant (*χ*^2^ = 12.845, *p* = 0.002), with urban residents being more knowledgeable (75.4%) than those in suburban (42.9%) and rural (50.0%) areas. Additionally, monthly income was significantly associated with CRS knowledge (*χ*^2^ = 10.922, *p* = 0.004), with respondents earning between 5,000 and 15,000 SAR per month having the highest level of awareness (85.7%) compared with respondents in lower- and higher-income groups. However, age (*χ*^2^ = 2.385, *p* = 0.665) and gender (*χ*^2^ = 0.18, *p* = 0.671) were not significantly associated with CRS knowledge, indicating that awareness levels were relatively consistent across these categories.

### Logistic regression analysis showing the relationship between CRS knowledge and independent influencers

The logistic regression analysis examined the associations between CRS knowledge and sociodemographic influencing factors and found several significant predictors (Table 2). Language was a key factor, with Arabic speakers being less likely than English speakers to lack CRS knowledge. The odds ratio (OR) for Arabic speakers was 0.21 (*p* = 0.010), indicating they were about 79% less likely to be unaware of CRS knowledge than their English-speaking counterparts, with a 95% CI of 0.06–0.69.

**Table 2:** Logistic regression analysis showing the relationship between CRS knowledge and independent influencing factors.

Dependent: good knowledge of CRS					
Independent influencers	Estimated coefficient (B)	Standard error (S. E.)	*p*-value	Adjusted Odds Ratio (AOR)	95% C. I. for AOR
Language – Arabic	−1.56	0.6	**0.01**	0.21	0.06–0.69
English	Ref				
Educational level			**0.035**		
High school	1.1	0.64	0.088	2.99	0.85–10.53
College/University	0.08	0.54	0.879	1.09	0.38–3.14
Postgraduate	Ref				
Occupation			**0.032**		
Students in the health field	0.76	0.84	0.362	2.14	0.42–11.02
Students (non-medical)	0.44	0.9	0.626	1.55	0.27–8.96
Education sector Job	1.09	0.99	0.269	2.98	0.43–20.58
Government employee	−0.85	0.98	0.386	0.43	0.06–2.92
Healthcare worker	0.63	1.07	0.557	1.87	0.23–15.29
Retired	1.01	1.29	0.432	2.75	0.22–34.43
Unemployed	Ref				
Place of residence			**0.005**		
Urban	1.21	0.81	0.137	3.35	0.68–16.46
Suburban	−0.28	0.94	0.766	0.76	0.12–4.76
Rural	Ref				
Monthly income (in SAR)			**0.017**		
Less than 5,000 per month	−0.08	0.39	0.839	0.92	0.43–2
5,000–15,000 per month	1.06	0.44	**0.017**	2.88	1.21–6.86
More than 15,000 per month	Ref				
Constant	−1	1.32	0.451	0.37	

Bold values indicate statistically significant results (*p* < 0.05).

Educational level was significantly associated with overall CRS knowledge (*p* = 0.035). Although none of the individual educational categories reached statistical significance on their own, respondents with a high school education had an OR of 2.99 (*p* = 0.088), indicating a trend toward greater knowledge. In contrast, those with a college/university education had an OR of 1.09 (*p* = 0.879) compared to postgraduates (reference group).

The respondents’ occupational role was also a significant predictor (*p* = 0.032). While individual factors, such as being a student in the health field (OR = 2.14, *p* = 0.362) or in the education sector (OR = 2.98, *p* = 0.269), were linked to higher odds of CRS knowledge, these factors alone did not reach statistical significance. However, the overall significance of the function variable suggests that it is an important factor when considered together.

The place of residence was significantly associated with CRS knowledge (*p* = 0.005). Although the OR for urban residents was 3.35 (*p* = 0.137), indicating that urban residents were more likely to be aware of CRS than rural residents, this association was not statistically significant. Monthly income also had a significant overall effect (*p* = 0.017). Notably, respondents earning between SAR 5,000 and 15,000 per month were significantly more likely to be knowledgeable about CRS than those earning more than SAR 15,000, with an OR of 2.88 (p = 0.017; 95% CI: 1.21–6.86). Income below SAR 5,000 did not differ significantly from the highest income group (OR = 0.92, *p* = 0.839).

In conclusion, language, place of residence, education level, functional role, and monthly income significantly influenced respondents’ knowledge of the CRS, with Arabic, mid-range income, and specific educational and occupational profiles associated with greater awareness.

### Principal component analysis for knowledge about common symptoms of CRS

The data were suitable for PCA, with the KMO measure indicating adequate sampling adequacy (=0.631) and a statistically significant Bartlett’s test of sphericity (*χ*^2^ = 155.848, df = 28, *p* < 0.001). The retained four components (eigenvalues > 1) explained 63.8% of the total variance. PCA of knowledge about common CRS symptoms identified four components that together reflected how respondents conceptually grouped symptoms (Table 3). Component 1 captured a systemic–infectious pattern, with high loadings for fever (0.792), cough (0.582), nasal discharge (0.496), and fatigue (0.495). These symptoms are commonly associated with generalized infection or inflammation, suggesting that respondents with high scores on this component recognize CRS as part of a broader systemic illness. Component 2 represented a facial–sensory dimension, dominated by facial pain or pressure (0.760) and loss of smell, consistent with more sinus-specific and neurologically linked manifestations. Component 3 reflected neurological and fatigue-related symptoms, mainly headache (0.701) and fatigue (0.678). This component may indicate an understanding of less obvious or secondary symptoms of CRS, particularly those affecting quality of life and mental well-being. Component 4 was characterized by nasal congestion (0.813) and discharge (0.411), aligning with core nasal symptoms that define CRS.

**Table 3:** Principal component analysis for knowledge about common symptoms of CRS.

Responses	Component 1	Component 2	Component 3	Component 4
Nasal congestion	0.129	0.217	0.176	0.813
Facial pain or pressure	0.058	0.76	0.059	0.026
Nasal discharge	0.496	−0.198	−0.201	0.411
Cough	0.582	0.378	−0.231	−0.36
Fatigue	0.495	−0.208	0.678	0.068
Headache	−0.29	0.005	0.701	−0.241
Fever	0.792	0.004	0.121	−0.249
Loss of sense of smell	−0.135	0.687	0.148	0.054

Overall, the PCA demonstrated that respondents’ knowledge of CRS symptoms clustered into distinct domains, including core nasal symptoms, facial and sensory symptoms, systemic manifestations, and neurological or fatigue-related features. These components indicate that while primary CRS symptoms were well recognized, awareness of secondary and nonspecific symptoms was more fragmented.

### Principal component analysis for knowledge about the risk factors of CRS

The PCA of CRS risk factor knowledge revealed three components (Table 4). Component 1 represented a broad environmental and structural risk dimension, with high positive loadings for smoking (0.662), air pollution (0.571), structural abnormalities (0.534), allergies (0.467), and viral infection (0.423). These factors are commonly recognized as environmental or external contributors to CRS, indicating that this component reflects a general understanding of common and modifiable risks.

**Table 4:** Principal component analysis for knowledge about the risk of CRS.

Responses	Component 1	Component 2	Component 3
Allergies	0.467	−0.336	0.491
Air pollution	0.571	−0.466	−0.039
Asthma	0.201	0.796	0.233
Viral infection	0.423	−0.191	−0.294
Structural abnormalities (such as a deviated septum)	0.534	0.126	−0.061
Smoking	0.662	0.346	0.161
Genetic	−0.261	−0.183	0.78

Component 2, centered on respiratory comorbidities, was primarily influenced by asthma (0.796) and, to a lesser extent, by smoking (0.346), suggesting that some respondents associated CRS with chronic airway disease rather than with local sinonasal factors alone. Component 3 reflected awareness of genetic and immunologic factors, primarily driven by genetic predisposition (0.780) and secondarily by allergies (0.491). This component may reflect an understanding of hereditary and immune-related risk factors, acknowledging internal or inherited predispositions to CRS, although genetic factors were the least frequently recognized overall in the dataset.

Collectively, the PCA results show that respondents mainly understood CRS risk as related to environmental and modifiable factors, with much less awareness of genetic or immunologic predispositions. This gap highlights an important educational need to improve understanding of non-environmental factors contributing to CRS.

### Principal component analysis for knowledge about CRS complications

The PCA of knowledge about CRS complications revealed two main components, indicating how respondents distinguished among possible complications (Table 5). Component 1 identified a pattern of severe systemic–neurological complications, with high loadings for orbital abscess (0.812), intracranial complications (0.739), meningitis (0.665), and vision problems (0.594). These conditions represent serious outcomes of untreated or poorly managed CRS that can extend beyond the sinuses, indicating a spectrum of responses that recognize the potential for critical medical issues. Component 2 reflected a more localized ocular aspect, driven primarily by an eye socket abscess (0.826) and supported by vision problems (0.570), suggesting partial awareness of orbit-focused complications distinct from broader neurological risks.

**Table 5:** Principal component analysis for knowledge about CRS complications.

Responses	Component 1	Component 2
Eye socket abscess	0.01	0.826
Intracranial complications	0.739	0.318
Meningitis	0.665	−0.415
Orbital abscess	0.812	−0.376
Vision problems	0.594	0.57

In summary, the PCA showed that respondents’ knowledge of CRS complications tends to fall into two categories: (1) a broader understanding of serious systemic or neurological risks and (2) awareness of localized eye-related issues. This insight could help tailor public health messages to improve overall awareness of both local and severe systemic outcomes of CRS.

### Descriptive analysis of attitudes and practices related to CRS

A descriptive analysis of respondents’ attitudes and practices regarding CRS revealed their perceptions, beliefs, and self-reported behaviors (Table 6). Most participants (67.5%) correctly recognized that CRS is not contagious; however, a substantial proportion remained uncertain, indicating residual misconceptions. Awareness of CRS within personal networks was moderate, with 38.0% reporting familiarity with individuals affected by CRS. Regarding perceived disease severity, 58.7% considered CRS somewhat serious, whereas 26.4% regarded it as a serious health condition.

**Table 6:** Descriptive analysis of attitudes and practices related to CRS.

Attitude and practice	No.	%
CRS is a contagious disease		
No	245	67.50%
Not Sure	92	25.30%
Yes	26	7.20%
Know about anyone in the circle who has been diagnosed with CRS		
No	225	62.00%
Yes	138	38.00%
How serious CRS as a health issue		
Not serious	46	12.70%
Somewhat serious	213	58.70%
Serious	96	26.40%
Very serious	8	2.20%
Want to Seek medical help if you have symptoms of CRS?		
Not Likely	25	6.90%
Somewhat likely	97	26.70%
Likely	151	41.60%
Very likely	90	24.80%
Think CRS can be effectively managed or treated.		
No	27	7.40%
Not Sure	165	45.50%
Yes	171	47.10%
How do lifestyle factors influence the development of CRS?		
No effect	9	2.50%
Weak effect	9	2.50%
Medium effect	104	28.70%
Great effect	196	54.00%
Not Sure	45	12.40%
How important is it to increase public awareness about CRS?		
Important	167	46.00%
Not important	7	1.90%
Somewhat important	61	16.80%
Very important	128	35.30%
CRS may require long-term medical treatment.		
Agree	148	40.80%
Disagree	41	11.30%
Neutral	95	26.20%
Strongly agree	70	19.30%
Strongly disagree	9	2.50%
Surgery may sometimes be necessary to treat CRS.		
Strongly agree	55	15.20%
Agree	116	32.00%
Neutral	136	37.50%
Disagree	40	11.00%
Strongly disagree	16	4.40%
Regular follow-up with an otolaryngologist is essential.		
Strongly agree	124	34.20%
Agree	137	37.70%
Neutral	91	25.10%
Disagree	7	1.90%
Strongly disagree	4	1.10%
Tools of Prevention of CRS		
Strongly agree	149	41.00%
Agree	123	33.90%
Neutral	68	18.70%
Disagree	17	4.70%
Strongly disagree	6	1.70%
Tools can significantly reduce the risk of developing CRS		
Strongly agree	134	36.90%
Agree	130	35.80%
Neutral	81	22.30%
Disagree	15	4.10%
Strongly disagree	3	0.80%
CRS can significantly affect the quality of life		
No	25	6.90%
Not sure	93	25.60%
Yes	245	67.50%
Using surgery to treat CRS		
Strongly support	14	3.90%
Support	109	30.00%
Neutral	152	41.90%
Oppose	54	14.90%
Strongly oppose	34	9.40%
The first action if diagnosed with CRS		
Consult a doctor	288	79.30%
Do nothing	11	3.00%
Self-medication	34	9.40%
Use home remedies	30	8.30%
Think about alternative medicine or herbs.		
No	93	25.60%
Not sure	167	46.00%
Yes	103	28.40%

Health-seeking behaviors were generally positive, with 66.4% of respondents reporting they were likely or very likely to consult a physician if symptoms occurred. Perceptions of treatment effectiveness were mixed: 47.1% believed CRS could be effectively managed, whereas 45.5% were uncertain. Lifestyle factors were widely acknowledged: 54.0% indicated that lifestyle strongly influences CRS development, and an overwhelming majority (90.1%) emphasized the importance of public awareness initiatives.

Long-term disease management was recognized by many respondents: 60.1% agreed or strongly agreed that CRS may require prolonged treatment. Willingness to consider surgical intervention was moderate (47.2%), while regular follow-up with an otolaryngologist was strongly supported (71.9%). Preventive approaches were also widely endorsed: 74.9% supported preventive measures in principle, and 72.7% affirmed their effectiveness.

The impact of CRS on quality of life was well recognized, with 67.5% agreeing that the condition can significantly affect daily functioning. Upon diagnosis, most respondents (79.3%) indicated they would seek medical care; however, 28.4% expressed openness to herbal or traditional remedies. Overall, these findings reflect generally appropriate attitudes and practices, alongside persistent uncertainties regarding contagiousness, treatment effectiveness, and alternative therapies, highlighting opportunities for targeted education and awareness efforts.

## Discussion

The findings of this study should be interpreted within the context of a regionally based sample that included a substantial proportion of health sciences students, reflecting the study’s focus on both current and future healthcare providers rather than exclusively on practicing clinicians. The results reflect the demographic characteristics of the Hail region in Saudi Arabia and provide a culturally relevant framework for assessing CRS awareness and knowledge. The vast majority of participants were Arabic-speaking. Compared with Western English-speaking countries, where CRS prevalence is estimated at 10.9% in Europe and 11.9% in the United States (7, 28), the burden of CRS in Saudi Arabia is considerably higher, with a reported national adult prevalence of 22.5% (10). Notably, Jafar et al. (22) reported that CRS with nasal polyps accounted for 67.7% of diagnosed cases, suggesting distinct epidemiological patterns within Arabic-speaking populations.

Most participants were young adults, college-educated, and urban residents, with males comprising 65.0% of the sample. Females were nevertheless adequately represented (35.0%), and no significant gender-based differences in CRS knowledge were observed, indicating that the modest male predominance did not materially influence knowledge patterns. Over half of the respondents were health sciences students, and 19.3% were current smokers, aligning with national data linking CRS risk to occupational exposure and secondhand smoke (29). Socioeconomic factors further shape smoking behavior, as individuals with higher income levels tend to smoke less (30), a pattern also observed here, with smoking more prevalent among middle- and low-income groups. Smoking cessation efforts are reportedly more common among older, higher-income males (31), and awareness of and use of cessation clinics are higher among urban and educated populations (32). Together, these findings highlight the need for public health strategies tailored to specific demographic and socioeconomic contexts to mitigate smoking-related CRS risk.

Recognition of cardinal CRS symptoms, such as nasal congestion, nasal discharge, and facial pain or pressure, was generally strong, indicating that participants could identify the classic clinical presentation of the disease. This aligns with symptom prevalence reported in clinical populations (33–35). However, awareness declined as symptom specificity decreased. Systemic and secondary manifestations, including cough, loss of smell, fatigue, and fever, were less consistently recognized, suggesting that the broader systemic impact of CRS may be underappreciated. Most notably, the headache that was reported by a majority of CRS patients (34) was rarely identified, indicating a substantial knowledge gap. This lack of recognition of nonspecific but common symptoms that significantly affect quality of life may contribute to delayed diagnosis and incomplete disease management.

A similar imbalance was observed in awareness of CRS risk factors. Participants demonstrated strong recognition of environmental and acquired contributors, such as allergies, air pollution, and smoking, whereas awareness of genetic predisposition was notably limited. This pattern aligns with established evidence identifying smoking (36) and comorbid asthma (37) as major contributors to CRS, suggesting that public health messaging around modifiable risk factors has been effective. In contrast, the limited recognition of genetic susceptibility highlights a critical knowledge deficit that warrants targeted educational interventions.

Patterns of awareness regarding CRS-related complications further reinforce this imbalance. While visual disturbances were the most frequently recognized complication, awareness declined sharply for more severe and potentially life-threatening orbital and intracranial complications. This is concerning, as these complications are well-documented in the literature. Orbital complications commonly arise from sinus infections (38), and a substantial proportion of orbital cellulitis cases progress to abscess formation (39). The limited recognition of these serious outcomes, particularly among participants with healthcare backgrounds, highlights a gap in clinical preparedness that could delay urgent diagnosis and intervention.

Healthcare professionals were the primary reported source of CRS-related information, followed by digital platforms. This finding is consistent with the work of Ulloa et al. (26), who demonstrated that online support communities enhance knowledge and decision-making among CRS patients, with nearly 87% reporting improved understanding through digital engagement. These findings underscore the complementary role of online resources alongside traditional healthcare education. Overall, participants demonstrated generally appropriate attitudes and practices regarding CRS management, particularly in relation to healthcare-seeking behavior, specialist consultation, and preventive strategies. However, persistent uncertainty regarding treatment effectiveness and the role of alternative medicine suggests the need for improved educational interventions addressing misconceptions and evidence-based management approaches.

The PCA provided further insight into how participants conceptualized CRS complications, revealing two broad patterns: systemic–neurological threats and localized orbital sequelae. The clustering of intracranial complications within one component suggests that a subset of respondents recognized CRS as a potential source of severe morbidity, consistent with reported risks of meningitis, brain abscess, and cavernous sinus thrombosis (40–42). The second component emphasized ocular complications, reflecting awareness of disease extension from the sinuses to the orbit, which can result in optic neuropathy and permanent vision loss (43–45). Although this separation indicates some conceptual understanding of disease progression, overall recognition of specific high-risk complications, particularly orbital abscess, remained low. Because management of these outcomes depends on early recognition, advanced imaging (CT/MRI), and timely endoscopic intervention (25, 46), these findings highlight areas that urgently need targeted education. The PCA results should be interpreted as exploratory, as they were derived from self-reported categorical knowledge items and intended to describe awareness patterns rather than establish definitive latent constructs.

Although some participants showed a detailed understanding of CRS complications, overall recognition, especially of orbital abscesses and intracranial complications, remained limited. This highlights the need for targeted educational initiatives that address not only the existence but also the severity and clinical implications of CRS-related complications. These interventions are especially important in pediatric populations, where disease progression may be more aggressive and outcomes more severe (42, 47).

Overall, participants demonstrated generally proactive and health-conscious attitudes toward CRS, including willingness to seek medical care, recognition of lifestyle influences, and support for preventive measures and regular follow-up. Nonetheless, uncertainty persisted regarding the contagiousness of the disease, the effectiveness of treatment, and the role of alternative medicine. Consistent with prior qualitative studies (25, 46, 48), respondents acknowledged the substantial impact of CRS on quality of life and expressed concerns about the adequacy of available treatments. Although participants, predominantly healthcare professionals and students, reported readiness to seek care, existing literature indicates that patients frequently encounter barriers in primary care, including limited access, fragmented services, and dissatisfaction with provider communication (25, 46). Moreover, while long-term medical therapy and surgical interventions were generally accepted in this study, persistent symptoms and treatment-related frustration are commonly reported in real-world settings (48, 49). Earlier research has similarly emphasized the need for holistic care, enhanced patient education, and shared decision-making, findings that align with the persistent educational gaps observed even among medically informed individuals in the present study (25, 49). Collectively, these results underscore the importance of patient-centered approaches, improved provider communication, and evidence-based education to optimize long-term management and outcomes of CRS. The present study identified several sociodemographic factors associated with CRS knowledge, including language, education, occupation, place of residence, and monthly income. These variables were identified through logistic regression analysis and represent factors associated with knowledge rather than causal determinants of CRS awareness.

### Limitations of the present study

This study has certain limitations that should be considered when interpreting the findings. First, the study was conducted in a single region (Hail) using a convenience sampling approach, with a predominance of urban participants (92%), which may introduce geographic and selection bias and limit the generalizability of the findings beyond this setting. Additionally, response rate data were not captured, which restricts the ability to assess potential non-response bias.

Second, the sample primarily consisted of health sciences students, with practicing healthcare professionals accounting for only 5.0% of respondents. While this composition reflects the study’s intentional focus on future healthcare providers, it limits the applicability of the findings to the broader population of practicing clinicians and precludes robust comparisons across occupational subgroups. Therefore, the results should be interpreted as a regional assessment of CRS awareness among healthcare trainees and early-career professionals rather than a nationally representative evaluation. Moreover, detailed stratification of students according to academic year was not performed, which may have limited assessment of differences in CRS knowledge according to stage of training.

Although convenience sampling in a single-center study may not fully capture the national distribution of demographic and occupational characteristics, the sample included representation from both sexes, and no significant gender-based differences in CRS knowledge were observed. Third, because data were collected via self-administered questionnaires, the findings may be subject to recall and response biases, including potential over- or underestimation of knowledge and practices. Fourth, cross-sectional design limits the ability to infer causal relationships between sociodemographic factors and CRS-related knowledge, and the observed associations should be interpreted with caution.

Finally, although the questionnaire was developed based on established CRS guidelines and prior A-KAP literature and underwent expert review and pilot testing with acceptable internal consistency, it has not been fully validated using advanced psychometric methods (e.g., construct validity or confirmatory factor analysis). Consequently, measurement error and residual misclassification of knowledge and attitude domains cannot be excluded, and the instrument should be considered an early-stage tool requiring further validation. In addition, the PCA was exploratory in nature and based on categorical knowledge items, which may affect the stability, reproducibility, and generalizability of the identified components. Future research using larger, multicenter, and more diverse samples, along with fully validated instruments, is warranted to confirm and extend these findings.

## Conclusion

This study provides regionally relevant insights into A-KAP related to CRS among healthcare trainees and a smaller subgroup of practicing healthcare professionals in the Hail region of Saudi Arabia. While overall awareness was moderate and cardinal symptoms were generally well recognized, important knowledge gaps were identified, particularly regarding complications, genetic risk factors, and associated comorbidities. Sociodemographic factors, including language and income, were associated with variations in CRS-related knowledge; however, these findings should be interpreted with caution due to the cross-sectional design and sampling limitations. Participants generally demonstrated positive attitudes toward healthcare-seeking and support for preventive strategies, including specialist follow-up and public awareness initiatives, although uncertainties regarding treatment effectiveness and alternative therapies persisted. These findings underscore the need for targeted educational interventions, particularly within undergraduate and early clinical training, to address underrecognized aspects of CRS. Future multicenter studies with larger and more diverse samples are warranted to further validate these findings and assess their broader applicability.

## Data Availability

The original contributions presented in the study are included in the article/Supplementary material, further inquiries can be directed to the corresponding author.
