# Supplementary material for: Knowledge, attitudes, practices, and influencing factors associated with chronic rhinosinusitis among healthcare trainees and professionals in Hail, Saudi Arabia: a principal component analysis
Source: Front Public Health. 2026 Aug 14;14:1808606. doi: 10.3389/fpubh.2026.1808606 (PMC13522121; doi:10.3389/fpubh.2026.1808606)
Supplement: Supplementary file 1 [file Data_Sheet_1.PDF]

Knowledge, Attitudes, Practices, and Influencing Factors Associated with Chronic Rhinosinusitis among Healthcare Trainees and Professionals in Hail, Saudi Arabia: A Principal Component Analysis

Supplementary Tables: Principal Component Analysis (PCA) Results

Supplementary Table S1. Assessment of sampling adequacy and sphericity for Principal Component Analysis

Statistic	Value
KMO	0.631
Bartlett's Chi-square (χ^2)	155.848
degrees of freedom (df)	28
Significance (p-value)	<0.001

Supplementary Table S2. Initial and extracted communalities for the variables included in the Principal Component Analysis

Variable	Initial	Extraction
Nasal congestion	1.000	.756
Facial pain or pressure	1.000	.585
Nasal discharge	1.000	.495
Cough	1.000	.664
Fatigue	1.000	.752
Headache	1.000	.634
Fever	1.000	.704
Loss of sense of smell	1.000	.515

Supplementary Table S3. Initial eigenvalues and cumulative variance explained by the retained principal components

Component	Initial Eigenvalues			Extraction Sums of Squared Loadings		
	Total	% of Variance	Cumulative %	Total	% of Variance	Cumulative %
1	1.580	19.744	19.744	1.580	19.744	19.744
2	1.322	16.530	36.274	1.322	16.530	36.274
3	1.116	13.947	50.221	1.116	13.947	50.221
4	1.087	13.593	63.814	1.087	13.593	63.814
5	.875	10.943	74.757			
6	.784	9.801	84.558			
7	.714	8.930	93.488			
8	.521	6.512	100.000			

Extraction method: Principal Component Analysis. Components with eigenvalues >1 were retained.
